# HOXB6 down-regulation induced by retinoic acid pathway repression leads to chondrocyte proliferation inhibition and apoptosis in microtia

**DOI:** 10.1016/j.gendis.2024.101367

**Published:** 2024-06-25

**Authors:** Run Yang, Xin Chen, Siyi Wu, Chenlong Li, Ying Chen, Yaoyao Fu, Aijuan He, Duan Ma, Jing Ma, Tianyu Zhang

**Affiliations:** a ENT Institute, Department of Facial Plastic and Reconstructive Surgery, Eye & ENT Hospital, Fudan University, Shanghai 200031, China; b Key Laboratory of Metabolism and Molecular Medicine, Ministry of Education, Department of Biochemistry and Molecular Biology, School of Basic Medical Sciences, Fudan University, Shanghai 200032, China; c NHC Key Laboratory of Hearing Medicine (Fudan University), Shanghai 200031, China

Microtia, a congenital malformation affecting the external and middle ear, arises from disruptions during the development of embryonic branchial arches and cranial neural crest cells (CNCCs), making it the second most prevalent maxillofacial birth defect and a leading cause of conductive hearing loss.^1^ Aberrant gene expression is pivotal in microtia pathogenesis by influencing chondrocyte activities. Homeobox (HOX) genes stand out as regulators for proper skeletal patterning, and mutations within the HOX family have been implicated in microtia.^2^ Our transcriptomic microarray analysis of microtia revealed a substantial reduction in *HOXB6* expression in auricle cartilage.^3^ HOXB6, known for its crucial involvement in embryonic axis determination, when expressed ubiquitously in transgenic mice, resulted in microtia. However, the specific role of HOXB6 in microtia remains elusive. This study validates the down-regulation of HOXB6 in auricle cartilage obtained from microtia patients. We also postulate that the decrease observed in *HOXB6* expression levels, resulting from a reduction in retinoic acid (RA) pathway signaling, triggers the inhibition of chondrocyte proliferation and the promotion of apoptosis by directly governing the expression of targeted genes related to these cellular behaviors. This action leads to a decrease in the overall chondrocyte population, contributing to the phenotypes observed in microtia patients.

We initially confirmed the association between HOXB6 expression and microtia. Quantitative polymerase chain reaction (qPCR) and immunohistochemistry analysis revealed a significant decrease in HOXB6 mRNA and protein levels in auricle cartilage tissues sourced from microtia patients, respectively (Fig. 1A, B; Fig. S1A–C). These findings collectively provide evidence of a potential correlation between HOXB6 down-regulation and microtia.

**Figure 1:**
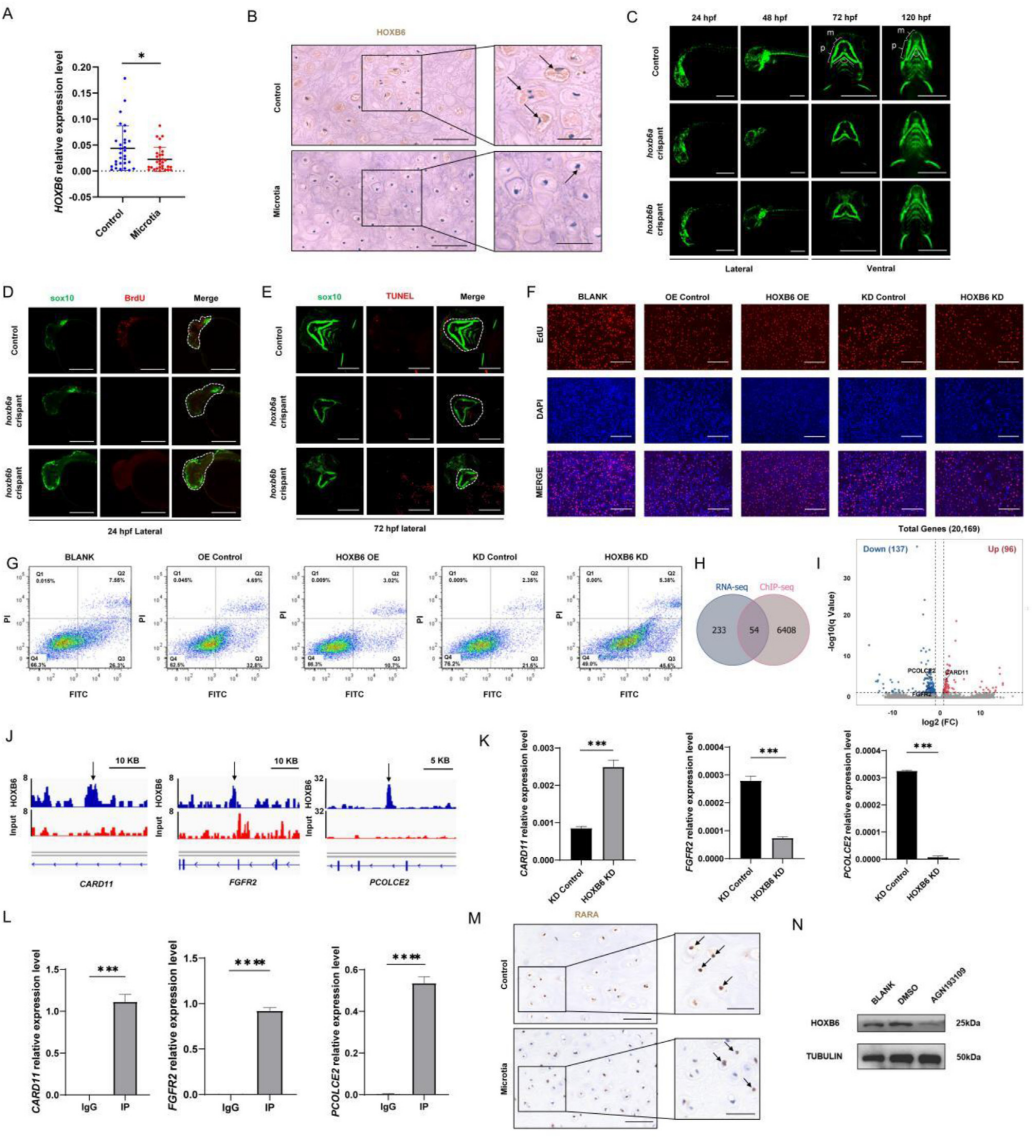
HOXB6 down-regulation induced by retinoic acid pathway repression leads to chondrocyte proliferation inhibition and apoptosis in microtia. **(A)** The mRNA levels of *HOXB6* in auricle cartilage tissues detected by qPCR assay in samples from patients with microtia (*n* = 30) and control (*n* = 30). ∗*P* < 0.05. **(B)** The protein level and localization of HOXB6 in the auricle cartilage tissue analyzed by IHC staining in low-power magnification (left, scale bar: 100 μm) and high-power magnification (right, scale bar: 50 μm). Arrows indicate HOXB6 staining. **(C–E)** Maximum projections of the confocal live image acquired from *hoxb6a* and *hoxb6b* crispants and controls. (C) Lateral views of *Tg*(*sox10:GFP*) zebrafish at 24 hpf and 48 hpf showing fluorescence intensity of CNCCs and ventral views of *Tg*(*sox10:GFP*) zebrafish at 72 hpf and 120 hpf showing CNCC-derived Meckel's cartilages (m), palatoquadrate cartilages (p), and angles between bilateral palatoquadrate cartilages. Scale bar: 500 μm. (D) Lateral views of zebrafish at 24 hpf, with proliferating cells labeled with BrdU, indicating CNCC proliferation. Scale bar: 300 μm. (E) Ventral views of zebrafish at 72 hpf, with apoptotic chondrocytes labeled with TUNEL. Dashed lines delineate the mandible area. Scale bar: 300 μm. **(F)** Immunofluorescence results from EdU assay indicating proliferation of HOXB6 overexpression (HOXB6 OE) or knockdown (HOXB6 KD) C28/I2 cells compared with control groups (OE Control, KD Control). *n* = 3 biologically independent experiments. **(G)** Flow cytometry analysis of cell apoptosis. C28/I2 cells were stained with Annexin V-FITC and PI. PI positive indicates cell necrosis; Annexin V positive indicates cell apoptosis; PI positive/Annexin V negative indicates cell necrosis (Q1); Annexin V positive/PI negative and Annexin V positive/PI positive indicate early apoptosis (Q3) and late apoptosis (Q2), respectively. **(H)** Intersection of RNA-seq and ChIP-seq results show 54 potential target genes of HOXB6. **(I)** Volcano plot shows the up-regulation of *CARD11* and down-regulation of *FGFR2* and *PCOLCE2* among the differentially expressed genes identified by RNA sequencing. **(J)** The peaks identified from the HOXB6 ChIP-seq show the overlapping with the enhancers (arrows) of *CARD11*, *FGFR2*, and *PCOLCE2*. **(K)** The qPCR assay shows the expression of *CARD11, FGFR2*, and *PCOLCE2* in HOXB6 down-regulated and control C28/I2 cells. *n* = 3 biologically independent experiments. **(L)** The binding of HOXB6 to *CARD11*, *FGFR2*, and *PCOLCE2* confirmed by ChIP-qPCR assay. *n* = 3 biologically independent experiments. Error bars display standard errors. ∗∗∗*P* < 0.001, ∗∗∗∗*P* < 0.0001. **(M)** The protein level and localization of RARA in the auricle cartilage tissue analyzed by IHC staining in low-power magnification (left, scale bar: 100 μm) and high-power magnification (right, scale bar: 50 μm). Arrows indicate RARA staining. **(N)** The western blots assay shows HOXB6 protein expression in C28/I2 cells treated with the RA antagonist AGN193109 (AGN) dissolved in DMSO for 120 h compared with DMSO treated controls (DMSO) and untreated controls (BLANK). IHC, immunohistochemistry; qPCR, quantitative real-time polymerase chain reaction. CNCC, cranial neural crest cells; hpf, hours post fertilization; mm, mandibular mesenchyme; p, palatoquadrate cartilages; m, Meckel's cartilage.

While zebrafish lacks auricles, it possesses mandibles derived from the same origin. To visualize the neural crest-derived mandible, we utilized a transgenic zebrafish line *Tg*(*sox10:GFP*) for rapid observation of phenotypes. The two homologous genes of *HOXB6* in zebrafish, namely *hoxb6a* and *hoxb6b*, exhibit elevated expression levels from 12 h post fertilization (hpf) to 48 hpf, aligning with the critical period of CNCC migration and pharyngeal arch development (Fig. S2C). We employed the CRISPR-Cas9 gene knockdown strategy to generate *hoxb6a* and *hoxb6b* mutant larvae (crispants), respectively (Fig. S2D). The overall development of crispants remains normal (Fig. S2E, F). Although we did not observe a difference in the number of CNCCs at 24 hpf (Fig. 1C; Fig. S2G), BrdU labeling revealed that the proliferation percentage of CNCCs in crispants is significantly reduced (Fig. 1D; Fig. S2M). As a result, at 48 hpf, the crispants showed a significant decrease in the number of CNCCs, especially in the mandibular mesenchyme (Fig. 1C, D). Furthermore, at 72 hpf and 120 hpf, we observed mandibular cartilage hypoplasia in crispants, characterized by significantly shortened Meckel's cartilages and palatoquadrate cartilages, as well as an augmented angle between the palatoquadrate cartilages, indicating palatoquadrate cartilage hypoplasia (Fig. 1C; Fig. S2I, L). This coincides with the increased apoptosis of mandibular chondrocytes in crispants at 72 hpf (Fig. 1E; Fig. S2N). These findings indicate that knocking down *hoxb6a* and *hoxb6b* in zebrafish leads to perturbed CNCC proliferation and chondrocyte apoptosis, ultimately contributing to the malformation of mandibular cartilage.

Subsequently, we down-regulated and up-regulated HOXB6 expression in C28/I2 cells (Fig. S3A–D). Both CCK8 and EdU assay demonstrated that down-regulation of HOXB6 could inhibit chondrocyte proliferation, while the overexpression of HOXB6 could promote this process (Fig. 1F; Fig. S3E–G). Additionally, flow cytometry revealed that down-regulation of HOXB6 accentuated chondrocyte apoptosis, whereas up-regulation of HOXB6 restrained the occurrence of apoptosis (Fig. 1G; Fig. S3H). However, changes in HOXB6 expression level did not affect the cell cycle of chondrocytes (Fig. S3I, J). These results underscore that HOXB6 plays a crucial role in regulating chondrocyte proliferation and apoptosis, highlighting its potential importance in the intricate orchestration of chondrocyte development and homeostasis within cartilage.

As a transcription factor, HOXB6 exerts its regulatory influence by binding to specific motifs of target genes. To explore the mechanisms underlying HOXB6's control over chondrocyte proliferation and apoptosis, we performed bulk RNA sequencing (RNA-seq) and chromatin immunoprecipitation sequencing (ChIP-seq) analysis of C28/I2 cells. The RNA-seq analysis revealed 233 differentially expressed genes (DEGs) between HOXB6 knockdown and control cell lines. Among these DEGs, 96 were significantly up-regulated, while 137 were significantly down-regulated (fold change ≥2, *P* value < 0.05) (Fig. S4A, B). Gene Ontology (GO) analysis illustrated that these DEGs were involved in bone morphogenesis, apoptosis cell clearance, and extracellular matrix formation (Fig. S4C). Kyoto Encyclopedia of Genes and Genomes (KEGG) analysis further highlighted the enrichment of DEGs in pathways associated with chondrogenesis (Fig. S4D). Concurrently, the ChIP-seq assays showed HOXB6 binding to 19,651 genomic regions. Overall, 49.13% of the HOXB6 peaks were located at intergenic regions, 1.96% at exons, 32.91% at introns, 4.23% at promoter regions, and 11.76% at upstream sequences of 6408 genes (Fig. S4E). The broken line plot (Fig. S4F) and the heatmap (Fig. S4G) showed the distance of the peak to the transcription start site in the FLAG-HOXB6 specific antibody and the input control group. The binding peaks revealed a putative HOXB6 binding motif of CUAAUU (Fig. S4H). Furthermore, GO term analysis of these predicted HOXB6 binding genes indicated their relevance to the regulation of intracellular signal transduction, cellular response to cytokine stimulus, and TOR signaling (Fig. S4I), while KEGG analysis revealed their involvement in pathways associated with necroptosis and pathways related to rheumatoid arthritis, a chondrocyte-related condition (Fig. S4J). By integrating the results of RNA-seq and ChIP-seq analysis, we identified 54 DEGs that were also the nearest genes identified around the HOXB6 ChIP-seq peaks (Fig. 1H). We hypothesized that these genes were target genes regulated by HOXB6. Among the top DEGs, we identified cell proliferation or apoptosis-related genes Caspase recruitment domain family member 11 (*CARD11*), fibroblast growth factor receptor 2 (*FGFR2*), and procollagen C-endopeptidase enhancer 2 (*PCOLCE2*) (Fig. 1I). The ChIP-seq peaks revealed HOXB6 binding to enhancer regions of these genes (Fig. 1J). Additional qPCR (Fig. 1K) and ChIP-qPCR (Fig. 1L) assays validated these findings, reaffirming the direct regulatory role of HOXB6 on these target genes. These results highlight that the impact of HOXB6 on chondrocytes is mediated by its control over genes associated with cell proliferation and apoptosis. The binding of HOXB6 to enhancer regions of these genes directly influences their expression, providing valuable insights into the molecular basis of microtia pathogenesis.

The RA signaling pathway has been reported to be related to microtia and regulates *HOXB6*.^1^ Using immunohistochemistry staining, we observed a decrease in the expression of RA receptor alpha (RARA) in the auricle cartilage of patients with microtia (Fig. 1M; Fig. S5A), and RARA serves as a marker of the RA signaling pathway. The qPCR results revealed a significant decrease in *RARA* mRNA expression in microtia cartilage (Fig. S5B). Meanwhile, from our published single-cell transcriptome data,^4^ we identified that another two genes involved in the RA signaling pathway, retinol binding protein 4 (*RBP4*) and all-trans retinoic acid induced differentiation factor (*ATRAID*), were significantly down-regulated in the auricle cartilage of microtia (Fig. S5C, D). To investigate the relationship between RA signaling and *HOXB6* expression level, by Western blot assay, we discovered that HOXB6 protein expression was significantly down-regulated after 120 h of treatment with the RA antagonist AGN193109^5^ at 100 nM in the cell culture medium (Fig. 1N).

In conclusion, our research suggests that HOXB6 may play a pivotal role in microtia and provide valuable insights into its molecular mechanisms.

## Ethics declaration

All methods were carried out in accordance with relevant guidelines and regulations established by the Institutional Research Ethics Committee of the Eye & ENT Hospital of Fudan University, China (Approval No. 2020069).

## Author contributions

Run Yang: conceptualization, formal analysis, data curation, methodology, investigation, writing - original draft, and writing - review & editing. Xin Chen: conceptualization, formal analysis, data curation, methodology, and investigation. Siyi Wu: formal analysis, data curation, methodology, and investigation. Chenlong Li: formal analysis and investigation. Ying Chen: formal analysis and investigation. Yaoyao Fu: investigation. Aijuan He: investigation. Duan Ma: project administration and resources. Jing Ma: project administration, funding acquisition, supervision, and writing - review & editing. Tianyu Zhang: project administration, funding acquisition, and supervision. All authors read and approved the manuscript.

## Conflict of interests

No conflict of interests is declared by the authors.

## Funding

This work was supported by the 10.13039/501100001809National Natural Science Foundation of China (No. 82271889, 82371173), the 10.13039/501100012166National Key Research and Development Program of China (No. 2021YFC2701000), the Natural Science Foundation Project of Shanghai Science and Technology Innovation Action Plan (China) (No. 23ZR1409400), and Shanghai Science and Technology Commission of China (No. 21DZ2200700).

## Data availability

All data are available upon request.

## Figures and Tables

**Figure S1:**
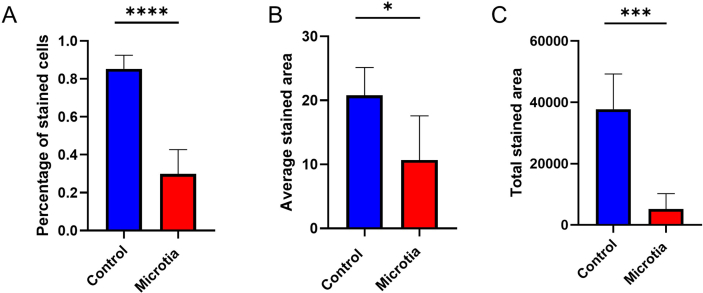
Quantification analysis of the immunohistochemistry results in Fig. 1B. **(A)** Percentage of HOXB6-stained cells. **(B)** Average HOXB6 stained area. **(C)** Total HOXB6 stained area. *P* values were from a two-tailed student's *t*-test. *n* = 3 biologically independent samples. Error bars display standard errors. ∗*P* < 0.05, ∗∗∗*P* < 0.001, ∗∗∗∗*P* < 0.0001.

**Figure S2:**
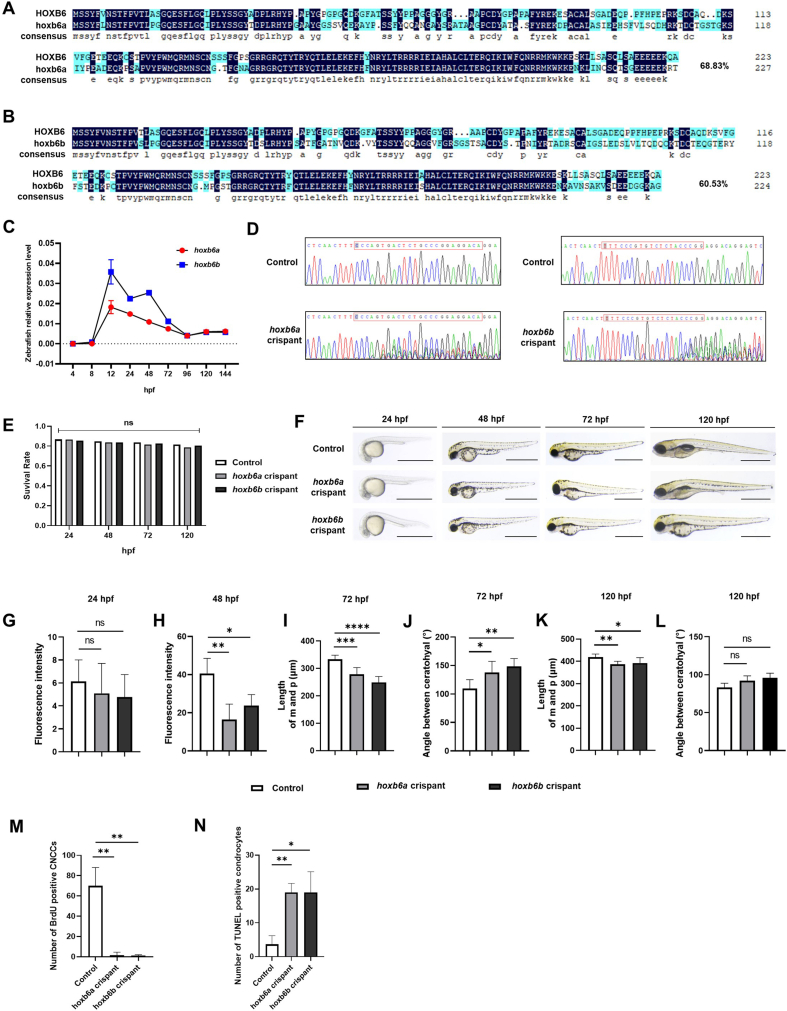
Construction and quantitative analysis of phenotypes in zebrafish with *hoxb6a* and *hoxb6b* disruption. **(A, B)** Amino acid sequence alignment of zebrafish hoxb6a (A) and hoxb6b (B) with mammalian HOXB6 protein. The similarity of hoxb6a was 68.83%, and that of hoxb6b was 60.53%. **(C)** The *hoxb6a* and *hoxb6b* mRNA expression assessed in 4 hpf to 144 hpf zebrafish by quantitative PCR (*n* = 3). **(D)** Sanger sequencing demonstrated that sgRNA (guide RNA) caused frameshift mutation at the targeted sites (red boxes). **(E)** Overall survival rates of *hoxb6a* and *hoxb6b* crispants and controls. *P* values were from a two-tailed student's *t*-test. ns, non-significant. **(F)** Lateral views of zebrafish from 24 hpf to 120 hpf. *n* = 3 larvae per group at each time point. Scale bar: 1 mm. **(G, H)** Quantification of sox10 fluorescence intensity at 24 hpf (G) and 48 hpf (H). **(I, L)** Length of Merkel's cartilage and palatoquadrate cartilage and angle between bilateral ceratohyal cartilages at 72 hpf and 120 hpf. *n* = 3 larvae per group at each time point. **(M)** Quantification of BrdU-positive proliferating CNCCs at 24 hpf. *n* = 3 larvae per group at each time point. **(N)** Quantification of TUNEL-positive apoptotic mandibular chondrocytes at 72 hpf. *n* = 3 biologically independent experiments. Error bars display standard errors. *P* values were from a two-tailed student's *t*-test. ∗*P* < 0.05, ∗∗*P* < 0.01, ∗∗∗*P* < 0.001, ∗∗∗∗*P* < 0.0001.

**Figure S3:**
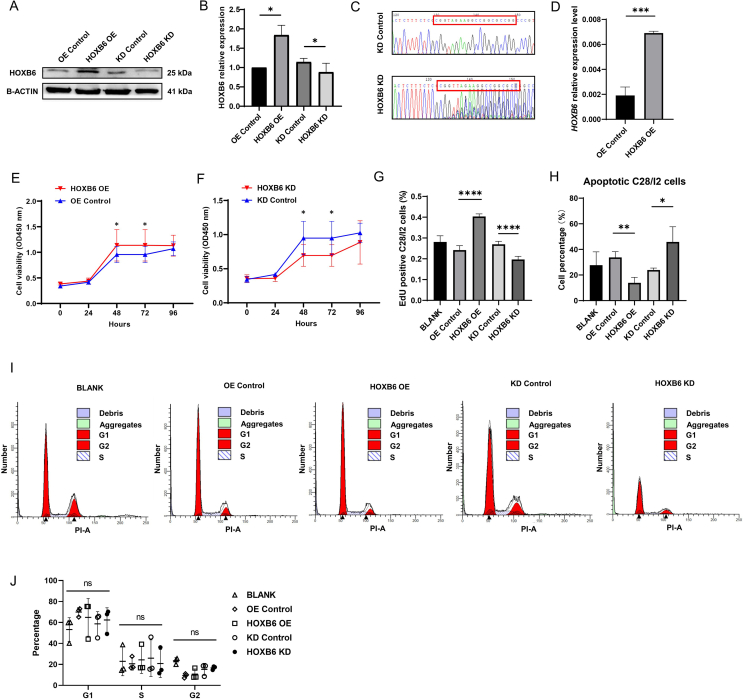
HOXB6 regulates the proliferation and apoptosis of chondrocytes but does not influence the cell cycle. **(A)** The efficiency of HOXB6 overexpression (HOXB6 OE) or knockdown (HOXB6 KD) C28/I2 cells compared with control groups (OE Control, KD Control) detected by western blot. **(B)** Gray-scale quantification of HOXB6 expression. *n* = 3 biologically independent experiments. **(C)** Sanger sequencing validated successful HOXB6 knockdown in C28/I2 cells. Guide RNA-targeted sequences (red box) caused frameshift in the *HOXB6* coding sequence. **(D)** Successful HOXB6 overexpression validated by quantitative PCR in C28/I2 cells. *n* = 3 biologically independent experiments. **(E, F)** CCK8 assay indicated the proliferation of HOXB6 OE (E) and HOXB6 KD (F) C28/I2 cells compared with control. *n* = 3 biologically independent experiments. **(G)** Quantification results from the EdU assay indicated the proliferation of C28/I2 cells compared with control. *n* = 3 biologically independent experiments. **(H)** Quantification results from flow cytometry assay indicated the apoptosis of C28/I2 cells compared with control. Cells were stained with Annexin V-FITC and PI. *n* = 3 biologically independent experiments. **(I, J)** Representative cell cycle flow cytometry results (I) and quantification (J) of cell percentage in G2/M phase (G2), S phase (S), and G0/G1 phase (G1). Error bars display standard errors. *P* values were from a two-tailed student's *t*-test. *n* = 3 biologically independent samples. ns, non-significant.

**Figure S4:**
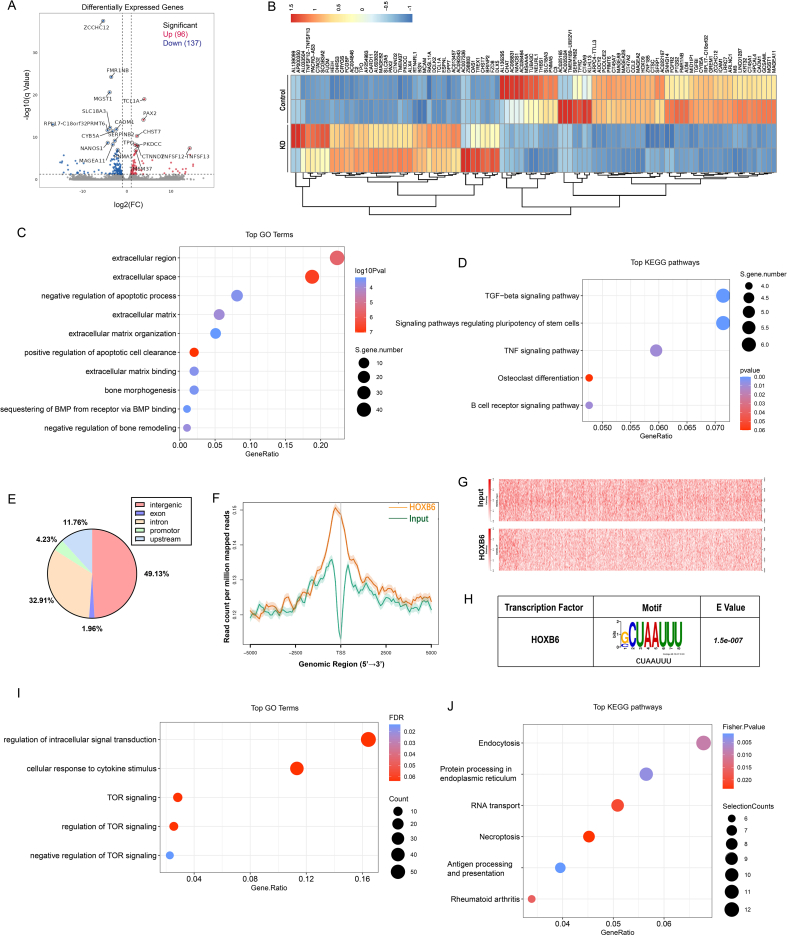
Analysis of the differentially expressed genes upon HOXB6 down-regulation and the identification of HOXB6 binding regions in C28/I2 cell lines. (A, B) RNA-seq volcano plot (A) and heatmap (B) show the differentially expressed genes (DEGs) (|log_2_FC| > 1, *Q* value < 0.05) in HOXB6 knockdown C28/I2 cells compared to the control group. (C, D) Top Gene Ontology (GO) terms (C) and Kyoto Encyclopedia of Genes and Genomes (KEGG) pathways (D) enriched in the differentially expressed genes (DEGs). (E) The pie chart categorizes the location of HOXB6-binding peaks across genomic regions. (F) Spike plot of the peak distribution within the distance of ±5 kb to the transcription start sites (TSS). (G) Heatmap of genome-wide HOXB6 accessibility compared with input control. (H) Annotated binding motif of HOXB6. The *E* value is the enrichment *P* value times the number of candidate motifs tested. The enrichment *P* value is calculated using Fisher's exact test for enrichment of the motif in the positive sequences. (I, J) Top Gene Ontology (GO) terms (I) and Kyoto Encyclopedia of Genes and Genomes (KEGG) pathways (J) enriched in the closest genes identified near the HOXB6 ChIP-seq peaks.

**Figure S5:**
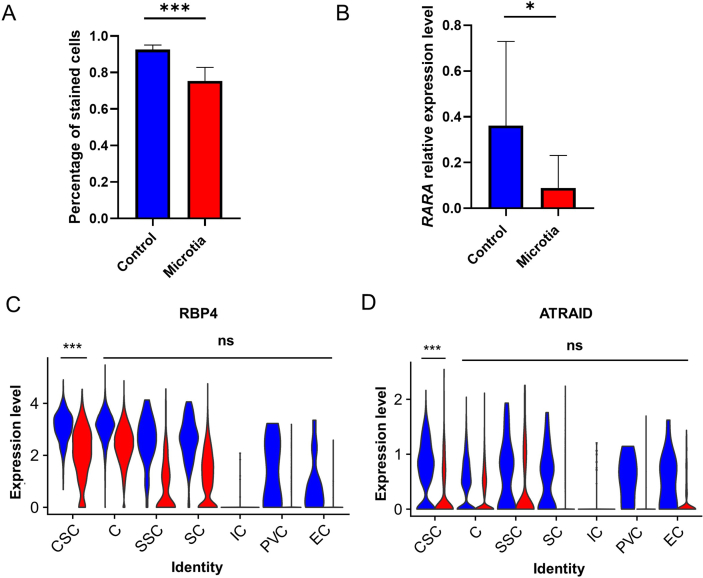
The effect of the retinoic acid signaling pathway on chondrocyte HOXB6 expression. **(A)** Quantification of the percentage of RARA-stained cells of immunohistochemistry results. *n* = 3 biologically independent samples. ∗∗∗*P* < 0.001. **(B)** The mRNA levels of *RARA* in auricle cartilage tissues detected by quantitative PCR assay in samples from patients with microtia (*n* = 11) and control (*n* = 11). ∗*P* < 0.05. **(C, D)** Violin plots display the *RBP4* (C) and *ATRAID* (D) expression in chondral stem/progenitor cells (CSC), chondrocytes (C), stromal stem/progenitor cells (SSC), stromal cells (SC), intermediate chondrocytes (ICs), perivascular cells (PVC), and early chondrocytes (EC) identified by single-cell transcriptome sequencing from our previous study.^4^ ∗∗∗*P* < 0.001.
