# Supplementary material for: HOXB6 down-regulation induced by retinoic acid pathway repression leads to chondrocyte proliferation inhibition and apoptosis in microtia
Source: Genes Dis. 2024 Jun 25;12(3):101367. doi: 10.1016/j.gendis.2024.101367 (PMC11786823; doi:10.1016/j.gendis.2024.101367)
Supplement: Multimedia component 1 [file mmc1.docx]

**SUPPLEMENTARY METHODS AND MATERIALS**

**Clinical samples**

In this study, we enrolled 30 patients diagnosed with third-grade unilateral isolated microtia and an equivalent number of 30 volunteers as controls for the quantitative real-time polymerase chain reaction (qPCR) assay. An additional group, consisting of 3 patients with third-grade unilateral isolated microtia and 3 volunteers, was recruited for immunohistochemistry (IHC) staining. The control group comprised volunteers undergoing tympanoplasty to address otitis media or cholesteatoma, and notably, they exhibited no auricle deformities or other concurrent disorders. Surgical resection of auricle cartilage tissues was performed at the Eye & ENT Hospital of Fudan University, and the collected auricle cartilage tissues were meticulously preserved using RNAlater RNA Stabilization Solution (Thermo Fisher Scientific, MA, USA) and subsequently stored at a temperature of -80°C to maintain RNA integrity for subsequent extraction. For IHC, tissues were fixed using 4% paraformaldehyde (Sinopharm Chemical Reagent, Shanghai, China).

**Ethics statement**

The acquisition of human tissue samples was conducted in strict adherence to the guidelines established by the Institutional Research Ethics Committee of the Eye & ENT Hospital of Fudan University, China (Approval No. 2020069). All procedures involving human participants were carried out in full compliance with the ethical principles outlined in the Declaration of Helsinki. The breeding and experimental protocols involving in zebrafish experiments were conducted in accordance with ethical standards reviewed and approved by the Ethics Committee of the Eye & ENT Hospital of Fudan University, China (Approval No. 2020069).

**RNA extraction, reverse transcription and quantitative polymerase chain reaction**

For the isolation of total RNA from human auricle cartilage, cells and zebrafish at 4, 8, 12, 48, 72, 96, 120, and 144 hours post fertilization (hpf), we employed the TRIzol reagent (Invitrogen, CA, USA) following the manufacturer's established protocol. Subsequently, we conducted reverse transcription utilizing the Hifair III 1st strand cDNA Synthesis SuperMix for qPCR reagent (Yeasen, Shanghai, China), supplemented with genome DNA digester (Yeasen, Shanghai, China) for synthesis of complementary DNA (cDNA). We performed qPCR on StepOnePlus™ Real-Time PCR System using SYBR® Green Realtime PCR Master Mix (TAKARA, Osaka, Japan). The qPCR reaction protocol comprised an initial pre-denaturation step at 95°C for 30 seconds, succeeded by 40 cycles, each involving denaturation at 95°C for 5 seconds, annealing at 60°C for 30 seconds, and extension at 95°C for 15 seconds. After amplification, melting curves were generated by maintaining the product at 60°C for 60 seconds, followed by a subsequent step at 95°C for 15 seconds. To assess relative expression levels of *HOXB6, PCOLCE2, FGFR2, CARD11, RARA*, *hoxb6a* and *hoxb6b*, we utilized the 2^-ΔΔCt^ method. This method involved normalization against the reference genes, human actin beta (*ACTB*) and zebrafish glyceraldehyde 3-phosphate dehydrogenase (*gapdh)*.

The primers used for qPCR were as follows:

*HOXB6*-Forward (F): TCTACCGCGAGAAAGAGTCG

*HOXB6*-Reverse (R): GGAGGAACTGTTGCACGAAT

*ACTB*-Forward (F): CCCTGGAGAAGAGCTACGAG

*ACTB*-Reverse (R): AGGTAGTTTCGTGGATGCCA

*PCOLCE2*-Forward (F): TACTTGGAAAATCACAGTTCCCG

*PCOLCE2*-Reverse (R): CGGCACAGGTTGTCACTCTC

*FGFR2*-Forward (F): AGTGCTTAATGCCCCTATGCT

*FGFR2*- Reverse (R): GCCCCTTGGTATGTAGAATGTCC

*CARD11*-Forward (F): AGTGCTTAATGCCCCTATGCT

*CARD11*- Reverse (R): GCCCCTTGGTATGTAGAATGTCC

*RARA*-Forward (F): AAGCCCGAGTGCTCTGAGA

*RARA*- Reverse (R): TTCGTAGTGTATTTGCCCAGC

*hoxb6a*-Forward (F): TTCCTCCGGATACACAGACC

*hoxb6a*-Reverse (R): TCTTGCGATGATCCTGACTG

*hoxb6b*-Forward (F): TGAACTCATGTAATGGAATGCC

*hoxb6b*-Reverse (R): CCAGTTCAAGAGTCTGAAACC

*gapdh*-Forward (F): GTGGAGTCTACTGGTGTTTC

*gapdh*-Reverse (R): GTGCAGGAGGCATTGCTTACA

**Immunohistochemical staining of auricle cartilage**

IHC was conducted to elucidate the distribution and localization of HOXB6 within auricle cartilage tissues. The primary antibody used was anti-HOXB6 (Abcam, Cambridge, UK) applied at a dilution of 1:50 and anti-RARA (Affinity Biosciences, Jiangsu, China) applied at a dilution of 1:100. Following the primary antibody incubation, we used Rabbit Anti-Goat IgG H&L (HRP) (Abcam, Cambridge, UK) or Goat Anti-Rabbit IgG H&L (HRP) (Abcam, Cambridge, UK) applied at a dilution of 1:2000 as secondary antibodies.

**Zebrafish line and CRISPR/Cas9-mediated knockdown**

The *Tg*(*sox10:GFP*) transgenic AB zebrafish strain, which facilitates the visualization of CNCCs and neural crest-derived mandibles, was obtained from Nanjing YSY Biotech Company Ltd (Nanjing, China). Zebrafish embryos were obtained through natural spawning from adult zebrafish. For executing loss-of-function experiments targeting *hoxb6a* and *hoxb6b*, we employed a modified protocol derived from Varshney et al.^1^. In brief, single guide RNAs (sgRNAs) for inducing CRISPR/Cas9-mediated knockdown in zebrafish was designed using CRISPR scan ([http://www.crisprscan.org](http://www.crisprscan.org/)). We designed four distinct sgRNAs for each target gene and synthesized in Invitrogen (MA, USA) following the manufacturer’s guidelines.

The four sgRNAs used for *hoxb6a* editing was:

sgRNA-a1: GTCCTCCGGGCAGAGTCACTGGG

sgRNA-a2: TAAAGGGTCTGTGTATCCGGAGG

sgRNA-a3: CTGCGCCCTCGCCAGCATAGAGG

sgRNA-a4: GAGTTCATCCGTTGCATCCAAGG

The four sgRNAs used for *hoxb6b* editing was:

sgRNA-b1: TTTCCCGTGTCTCTACCCGGAGG

sgRNA-b2: AGACTCGCTGGTCCTAACCCAGG

sgRNA-b3: TGCCTGGCAGCACTGGCCGCAGG

sgRNA-b4: AAGAGTCTGAAACCGAGTGTAGG

Prior to the experimental, we separately injected each sgRNA to confirm their efficacy in inducing insertions or deletions. For the generation of crispant zebrafish (F0 mosaic zebrafish), we injected a combination of four sgRNAs at a concentration of 400 ng/μL for each gene, along with 1 nL of Cas9 protein, into the single-cell stage embryos. In the control group, only 1 nL of Cas9 protein was injected. Following injection, the zebrafish were cultivated at 28℃ in E3 medium (containing 5 mM NaCl, 0.17 mM KCl, 0.33 mM MgSO4, 0.33 mM CaCl2, and 0.01% methylene blue) supplemented with 0.003% 1-phenyl 2-thiourea to prevent pigmentation. Sanger sequencing was used to confirm the successful perturbation of the target genes through CRISPR/Cas9-mediated manipulation.

**Bromodeoxyuridine assay**

To assess zebrafish CNCCs proliferation, at 24hpf, we conducted a Bromodeoxyuridine (BrdU) assay on *hoxb6a*/*b* knockdown and control group. In brief, the embryos were incubated for 6 hours in E3 medium enriched with 10 mM BrdU. Following this incubation, the embryos were fixed in 4% paraformaldehyde for 4 hours. Then the embryos were treated with 0.2 N hydrochloric acid (Sinopharm Chemical Reagent, Shanghai, China) for 1 hour at room temperature. Immunostaining was carried out by exposing the fixed embryos to anti-BrdU antibody (Santa Cruz Biotechnology, CA, USA) at a dilution of 1:100 overnight at 4℃. The embryos were then washed with phosphate-buffered saline (PBS) before incubation with Goat Anti-Mouse IgG 488 secondary antibody (Yisheng, Shanghai, China) at a dilution of 1:200 overnight at 4℃.

**Terminal deoxynucleotidyl transferase dUTP nick end labeling experiment**

At 72 hpf, zebrafish embryos underwent a terminal deoxynucleotidyl transferase dUTP nick end labeling (TUNEL) assay to detect apoptotic chondrocytes. In brief, the embryos were gently fixed overnight at 4°C using 4% paraformaldehyde. Subsequently, the embryos were thoroughly rinsed with 0.1% PBS-Tween solution. We employed the In Situ Cell Death Detection Kit, TMR red (Roche, Mannheim, Germany) for the detection of apoptotic cells.

**Zebrafish imaging**

Zebrafish specimens were mounted in 80% glycerol (Sinopharm Chemical Reagent, Shanghai, China) in 35 mm Glass bottom dishes (Cellvis, CA, USA), and confocal microscopic images were obtained using a TCS SP8 confocal inverted microscope (Leica, Germany). The images were processed by ImageJ software (v.1.52) for quantification analysis.

**Cell culture**

Both C28/I2 and HEK-293T cell lines utilized in the study were obtained from Merck (Shanghai, China) and cultured in high glucose Dulbecco's Modified Eagle Medium (Biological Industries, Beit HaEmek, Israel) supplemented with 10% fetal bovine serum (Gibco, CA, USA), and 1% Penicillin-Streptomycin Solution (NCM Biotech, Jiangsu, China) at 37℃ with 5% CO_2_.

**Plasmid construction, transformation and transfection**

The pCDH-CMV-MCS-EF1-Puro-HOXB6 plasmid was acquired from GeneRay (Shanghai, China). Additionally, the lentiCRISPRv2 plasmid was constructed using the protocol provided by ZhangLab^2;3^. To ensure accuracy, both plasmids underwent sequencing to confirm their fidelity with the sequences cataloged in the National Center for Biotechnology Information (NCBI) database. For the lentiviral CRISPR/Cas9 editing, the sgRNA oligos were employed. The sequences used were as follows:

Forward: ACAAGGGCTTTGCCACTTC

Reverse: TAGACCGGAGTGGAGCACTT

The plasmids were transformed into competent NcmDH5α cells. Following successful transformation, the plasmids were extracted and subsequently transfected into HEK-293T cells using L-pei polyethylenimine linear, MW 40000 (Yeasen, Shanghai, China). The lentiviral particles produced were employed to infect C28/I2 cells. Following infection, the C28/I2 cells were subjected to selection by puromycin (Yeasen, Shanghai, China). The efficiency of HOXB6 knockdown was evaluated through Sanger sequencing and western-blot assay.

**Western-blot assay**

C28/I2 cells were harvested and lysed using radioimmunoprecipitation assay (RIPA) buffer (Yeasen, Shanghai, China), supplemented with 1% phenylmethanesulfonyl fluoride (PMSF) (Beyotime, Shanghai, China). Protein concentrations were determined using a Bradford Protein Assay Kit (Abcam, MA, USA), and equal amounts of proteins were separated using a 10% SDS-polyacrylamide gel electrophoresis (SDS-PAGE) system (NCM Biotech, Suzhou, China). The separated proteins were then transferred from the gel onto nitrocellulose membranes (Pall, NY, USA). After blocking the membranes with 8% skimmed milk at room temperature for 1 hour, they were subjected to overnight incubation at 4°C with the primary antibodies of anti-HOXB6 (Novus Biologicals, CO, USA) at 1:1000 dilution and anti-beta-tubulin (Proteintech, IL, USA) at 1:1000 dilution.

**Cell proliferation assay**

The assessment of cell proliferation was conducted utilizing the Cell Counting Kit-8 (CCK-8) (Yeasen, Shanghai, China) and the 5-Ethynyl-2'-deoxyuridine (EdU) analysis kit (RiboBio, GuangZhou, China). For CCK-8 assay, a total of 10,000 C28/I2 cells were seeded into each well of a 96-well plate and allowed to adhere for 6 hours. After specific time intervals (0, 24, 48, 72 hours), 10 µl of CCK-8 solution was added to each well and incubated at 37°C for 2 hours. Then we used a PerkinElmer EnSpire spectrophotometer (Waltham, MA, USA) to measure the absorbance at 450 nm. For the EdU assay, C28/I2 cells were exposed to 50 μM EdU for 2 hours. EdU-labeled proliferative cells were identified using Apollo® 567 fluorescent dye. Images of the labeled cells were captured using the EVOS M5000 Imaging System (Thermo Fisher Scientific, MA, USA), and the acquired images were subsequently analyzed using ImageJ software (v.1.52).

**Cell apoptosis assay**

The cell apoptosis assay was carried out using the Annexin V-FITC Apoptosis Detection Kit (Dojindo, Tokyo, Japan), following these steps: C28/I2 cells were harvested and suspended in 100 μL of binding buffer. The suspended cells were stained with 5 μL of Annexin V-FITC Conjugate and 5 μL of propidium iodide (PI) solution (Dojindo, Tokyo, Japan) before incubating for 15 minutes at room temperature. Flow cytometric analysis was conducted using FACSCelesta™ Flow Cytometers (BD Biosciences, CA, USA), and the acquired data was analyzed using FlowJo software (v.10.8).

**Cell cycle analysis**

C28/I2 cells were harvested and fixed by the addition of 75% ethanol (Sinopharm Chemical Reagent, Shanghai, China) at 4°C and incubated overnight. Post-fixation, the cells were treated with 10 mg/mL Ribonuclease A (Sigma-Aldrich, St. Louis, MO, USA) at 37°C for 30 minutes. Following Ribonuclease A treatment, the cells were stained with a solution containing 1 mg/mL PI (Sigma-Aldrich, MO, USA). Cell cycle distribution was analyzed using FACSCelesta™ Flow Cytometers, and the acquired data was further analyzed using the Modfit software (v.3.1).

**RNA sequencing and data analysis**

The total RNA of HOXB6 knockdown C28/I2 cells was extracted using TRIzol reagent (Thermo Fisher Scientific, MA, USA). The quantity and purity of the extracted total RNA were assessed using the Bioanalyzer 2100 and RNA 6000 Nano LabChip Kit (Agilent, CA, USA). Post-purification, the mRNA was fragmented using Magnesium RNA Fragmentation Module (New England Biolabs, MA, USA) at 94℃ for 5-7 minutes. The cleaved RNA fragments were then subjected to reverse transcription to generate cDNA by SuperScript™ II Reverse Transcriptase (Invitrogen, CA, USA). After cDNA library preparation, 2×150bp paired-end sequencing (PE150) was carried out using an Illumina Novaseq™ 6000 (LC-Bio Technology, Hangzhou, China) in accordance with recommended protocols. The generated sequencing reads were filtered by Cutadapt (https://cutadapt.readthedocs.io/en/stable/, v1.9) and sequence quality was verified using FastQC (<http://www.bioinformatics.babraham.ac.uk/projects/fastqc/>, 0.11.9) according to a modified protocol by Thompson et al^4^. The filtered reads were aligned to a reference genome, and genes with a false discovery rate (FDR) below 0.05 and an absolute fold change (FC) of ≥2.0 were identified as differentially expressed genes (DEGs). Gene Ontology (GO) enrichment analysis^5^ and Kyoto Encyclopedia of Genes and Genomes (KEGG) pathway enrichment analysis^6^ were performed on the DEGs.

**Chromatin immunoprecipitation assay**

C28/I2 cells expressing HOXB6-DYKDDDDK plasmid and control cells were crosslinked with 1%, followed by quenching in glycine solution. DNA (10 ng) was extracted from each sample and prepared for subsequent analysis. TruSeq Nano DNA Sample Prep Kit (Illumina, CA, USA) was utilized for DNA sample preparation and ligation, adhering to the manufacturer's instructions. Immunoprecipitation was carried out using primary antibodies: anti-DYKDDDDK antibody (Abcam, Cambridge, UK) and Rabbit IgG control Polyclonal antibody (ProteinTech, IL, USA). Sequencing was conducted on an Illumina NovaSeq 6000 (Illumina, CA, USA) using the NovaSeq 6000 S4 Reagent Kit (Illumina, CA, USA), following the manufacturer’s guidelines, and sequencing reads were aligned to the human genome using the BOWTIE software (v.2.2.7). Then, chromatin immunoprecipitation (ChIP)-enriched DNA samples were further validated using ChIP-Quantitative Polymerase Chain Reaction (ChIP-qPCR). This analysis focused on specific genomic regions of interest (binding sites) of the target protein (HOXB6-DYKDDDDK). The qPCR quantified the enrichment of DNA fragments associated with the protein, relative to the IgG control. Primers were designed for ChIP-qPCR targeting specific binding sites:

*PCOLCE2*-peak-Q-F: TTAACCCGTTAATACCACAGAA

*PCOLCE2*-peak-Q-R: AGATAATGAAGACGGAACCAAA

*CARD11*-peak-Q-F: TTCTGAGTCTCCAACGTCCAT

*CARD11*-peak-Q-R: GGAGGCCATTATCCTAAGTGAA

*FGFR2*-peak-Q-F: TGTGGGAGTGAATGGCTAAG

*FGFR2*-peak-Q-R: TTGGTGGGAGGGTATGTTTT.

**Statistical analysis**

All experimental samples were prepared in triplicate and data were normalized to corresponding control groups. Each experiment was repeated three times independently. GraphPad Software Inc. (La Jolla, CA, USA) was utilized for conducting the statistical analysis. Paired two-tailed Student’s t-test was employed for the statistical analysis. A significance threshold of *P* < 0.05 was employed to determine statistical significance.

**Reference for Supplementary Methods and Materials**

1. Varshney GK, Carrington B, Pei W, et al. A high-throughput functional genomics workflow based on CRISPR/Cas9-mediated targeted mutagenesis in zebrafish. *Nat Protoc.* 2016;11(12):2357-2375.

2. Sanjana NE, Shalem O, Zhang F. Improved vectors and genome-wide libraries for CRISPR screening. *Nat Methods.* 2014;11(8):783-784.

3. Shalem O, Sanjana NE, Hartenian E, et al. Genome-scale CRISPR-Cas9 knockout screening in human cells. *Science.* 2014;343(6166):84-87.

4. Thompson O, von Meyenn F, Hewitt Z, et al. Low rates of mutation in clinical grade human pluripotent stem cells under different culture conditions. *Nat Commun.* 2020;11(1):1528.

5. Gene Ontology C. The Gene Ontology resource: enriching a GOld mine. *Nucleic Acids Res.* 2021;49(D1):D325-D334.

6. Kanehisa M, Furumichi M, Sato Y, Ishiguro-Watanabe M, Tanabe M. KEGG: integrating viruses and cellular organisms. *Nucleic Acids Res.* 2021;49(D1):D545-D551.
